# Prognostic value and immunological role of *PD-L1* gene in pan-cancer

**DOI:** 10.1186/s12885-023-11267-6

**Published:** 2024-01-02

**Authors:** Yongfeng Wang, Hong Jiang, Liangyin Fu, Ling Guan, Jiaxin Yang, Jingyao Ren, Fangyu Liu, Xiangyang Li, Xuhui Ma, Yonghong Li, Hui Cai

**Affiliations:** 1 The First Clinical Medical College of Lanzhou University, Lanzhou, Gansu, 730000 China; 2 General Surgery Clinical Medical Center, Gansu Provincial Hospital, Lanzhou, Gansu, 730000 China; 3 Key Laboratory of Molecular Diagnostics and Precision Medicine for Surgical Oncology in Gansu Province, Gansu Provincial Hospital, Gansu, 730000 China; 4 NHC Key Laboratory of Diagnosis and Therapy of Gastrointestinal Tumor, Gansu Provincial Hospital, 204 Donggang West Road, Lanzhou, Gansu, 730000 China; 5 School of Stomatology, Lanzhou University, Lanzhou, Gansu, 730000 China

**Keywords:** *PD-L1*, Prognosis, Cancers, Bioinformatics analysis

## Abstract

**Objective:**

*PD-L1*, a target of immune checkpoint blockade, has been proven to take the role of an oncogene in most human tumors. However, the role of *PD-L1* in human pan-cancers has not yet been fully investigated.

**Materials and methods:**

Pan-cancer analysis was conducted to analyze expression, genetic alterations, prognosis analysis, and immunological characteristics of *PD-L1*. Estimating the correlation between *PD-L1* expression and survival involved using pooled odds ratios and hazard ratios with 95% CI. The Kaplan–Meier (K-M) technique, COX analysis, and receiver operating characteristic (ROC) curves were applied to the survival analysis. Additionally, we investigated the relationships between *PD-L1* and microsatellite instability (MSI), tumor mutational burden (TMB), DNA methyltransferases (DNMTs), the associated genes of mismatch repair (MMR), and immune checkpoint biomarkers using Spearman's correlation analysis. Also, immunohistochemical analysis and qRT-PCR were employed in evaluating *PD-L1’*s protein and mRNA expression in pan-caner.

**Results:**

*PD-L1* showed abnormal mRNA and protein expression in a variety of cancers and predicted prognosis in cancer patients. Furthermore, across a variety of cancer types, the aberrant *PD-L1* expression was connected to the MSI, MMR, TMB, drug sensitivity, and tumor immune microenvironment (TIME). Moreover, *PD-L1* was significantly correlated with infiltrating levels of immune cells (T cell CD8 + , neutrophil, and so on).

**Conclusion:**

Our study provides a better theoretical basis and guidance for the clinical treatment of *PD-L1*.

**Supplementary Information:**

The online version contains supplementary material available at 10.1186/s12885-023-11267-6.

## Introduction

Despite being one of the most feared diseases of the 20th century, cancer is still widespread in the 21st century [1]. Every fourth person has a lifelong risk of developing cancer, which is a shocking state of affairs [2]. As a powerful anticancer strategy, cancer immunotherapy using immunotherapeutics has aroused people's wide concern [3]. In the above process, immune checkpoint control plays a pivotal role, which has become a research hotspot [4]. One of the primary causes of cancer is the differential expression and action of immune checkpoint molecules. Repairing this immune checkpoint malfunction is therefore a crucial treatment approach for tumors [5]. For instance, some studies demonstrated that *PD-1* and *PD-L1* inhibitors effectively inhibit lung cancer and melanoma by causing tumor cells to undergo apoptosis by disrupting the *PD-1*/*PD-L1* signaling pathway [6, 7].

Immunotherapy is a method of treating disease by regulating the immune function by targeting the body's immune status [8]. At present, almost all patients with mNSCLC are treated with *PD-1* or *PD-L1* in the first-line setting, except for mNSCLC carrying targeted oncogenes [9]. Furthermore, the *PD-L1*’s correlation with immunotherapeutic response provides patients with the most promising choices of anti-gastric cancer drugs [10]. An immunotherapeutic drug stimulates the immune system and eliminates malignancies in cancer immunotherapy. Using immunosuppressive drugs known as immunological checkpoints, the body's immune activation can be controlled [11–13]. At present, the most commonly prescribed medications are immunotherapy inhibitors (*PD-L1*, *CDLA4*, *PD-1*, and so on) [14]. One of the biological characteristics of malignancy is that cells are able to evade immune response through different pathways like the *PD-1*/*PD-L1* pathway [15]. Among them, some kinases play important roles in the activation of *PD-L1* [16]. For instance, the overexpression of *CDC28* protein kinase regulatory subunit 1B (*CKS1B*) could promote cell viability and invasion of papillary thyroid carcinoma cells through activation of *STAT3*/*PD-L1* signaling and Akt phosphorylation [17]. When programmed death protein 1 binds to programmed death-ligand-1, it triggers a downstream signaling cascade that prevents T-cell activation and suppresses the immune system's capacity to mount any inflammatory response [18]. TNBC can develop in the context of *PD-1* overexpression in malignant cells because these cells are capable of dodging the immune system’s reaction and multiplying out of control [19–21]. Furthermore, relevant molecular dynamics simulations revealed that the docking time between *PD-L1* and the ligand was at least 300 nanoseconds [22]. Anti-*PD-1*/*PD-L1* immune checkpoint inhibitors (ICIs) can be used to stop the binding relationship between *PD-1*/*PD-L1* to halt this rapid growth and spread [23]. This will trigger a powerful immune response that will kill malignant cells [24].

*PD-1* and *PD-L1*’s combination can protect healthy body tissue by reducing the overreaction of autoimmunity and reducing the damage caused by an overactive immune response [25]. However, for tumor patients, *PD-1* and *PD-L1*’s combination can decrease the vitality and proliferation ability of T cells in the tumor microenvironment and lose the ability to normally identify or kill tumor cells, which will comparatively improve the propagation speed, proliferation ability, and invasion of tumor cells, as well as promote tumor cell metastasis [26]. *PD-L1*'s function in tumor immunity and its underlying mechanisms, however, are unknown.

A comprehensive analysis of 33 different cancer types was performed to further study the association between *PD-L1* (also known as *CD274*) and prognosis. Additionally, we investigated *PD-L1*'s potential roles in a variety of malignancies and found evidence that it may be a predictive biomarker and is strongly connected with immune infiltration for several tumors.

## Methods

### Data source and processing

Information about the sample and analysis of *CD274* expression in human pan-cancer. We obtained data on *CD274* expression in 31 normal tissues and 21 tumor cell lines from the Cancer Genome Atlas (TCGA), the Cancer Cell Line Encyclopedia (CCLE) database (https://portals.broadinstitute.org/ccle/), and the Genotype-Tissue Expression (GTEx) portal (https://gtexportal.org/home/). By merging information from TCGA and the GTEx database for normal tissues, the difference in *CD274* expression between cancer and normal tissues was examined [27]. To collect all TCGA cancers' mutation types, mutation sites, alteration frequency information, and 3D candidate proteins structure, cBioPortal was applied. Somatic mutations and clinical follow-up data for patients with 33 different kinds of cancer were gathered from the TCGA database, together with data from level 3 RNA sequencing. Quartile normalization and log_2_ transformation were used to normalize the expression levels.

### Mismatch Repair System (MMRS) and DNA methyltransferase analysis

Tumorigenesis may result from defects in the DNA MMRS [28]. The TCGA database was used to determine the mutation levels of 5 MMR genes (*EPCAM*, *MLH1*, *MSH6*, *MSH2*, and *PMS2*). DNMTs also have a significant impact on how chromatin structure and gene expression are altered [29]. This study employed Spearman correlation analyses to evaluate the connection between *CD274* expression and 5 MMR genes, as well as the 4 methyltransferases (DNMT3B, DNMT3A, DNMT2, and DNMT1) by using the R-packages “reshape2” and “RColorBrewer”.

### Survival and prognosis analysis

K-M analysis, univariate and multivariate COX regression analysis were used to evaluate the correlation between *CD274* gene expression and patients' prognosis in 33 different malignancies via forest plots and K-M curves. For clinicopathological correlation analysis, R-package “limma” and “ggpubr” were applied. As a next step, ROC curves were constructed using the surviving ROC package, and the predictive power was determined by calculating the area under the curve (AUC). Besides, calibration and nomogram plots were obtained by the RMS package (version 6.2–0) and survival package (version 3.2–10) for predicting 1-year, 3-year, and 5-year OS.

### Correlations between *CD274* expression and immune

From the TIMER database (https://cistrome.shinyapps.io/timer/), the scores of these 6 tumor-infiltrating immune cells (TIICs) (CD4 + T cells, CD8 + T cells, macrophages, B cells, dendritic cells, and neutrophils) in 33 tumors were obtained. 10,897 samples from the TCGA are available in the TIMER database. Additionally, using Spearman correlation analyses, we assessed the associations between *CD274* expression and TIICs, immunological checkpoint marker expression levels as well as immune/stromal scores. An estimation algorithm in R-package “estimation” and “limma” was applied to assess the matrix score and immune score of stromal cells and immune cells (*P* < 0.001 as a cut-off value) [30].

### Drug sensitivity analysis of *CD274*

To analyze *CD274* chemosensitivity in different tumors, CallMinerTM was applied to download NCI-60 compound activity data and RNA-seq expression files (https://discover.nci.nih.gov/cellminer/home.do). Some FAD or clinically approved drugs were chosen for analysis.

### Pathway analysis of *CD274*

Specifically, the study made use of gene sets that were downloaded from the Gene Set Enrichment Analysis (GSEA) website (https://www.gsea-msigdb.org/gsea/downloads.jsp). Gene Ontology (GO) and KEGG (Kyoto Encyclopedia of Genes and Genomes) were implemented for *CD274* annotation by R-package“org.Hs.eg.db”, “clusterProfiler”, and “enrichplot” [31]. From the KEGG pathway database (http://www.kegg.jp/kegg/kegg1.html) and the WikiPathways database (https://www.wikipathways.org/), significant biological pathways related to *CD274* were obtained and presented.

### Immunohistochemical analysis

Later on, the HPA (https://www.proteinatlas.org/) was utilized to present the human protein expression models in normal and tumor tissues, from which we can find special protein expressions that were differentially expressed in specific tumors. Herein, the expression pattern of *CD274* in normal and tumor tissues was obtained by means of immunohistochemistry images.

### Cell culture

Gastric cell lines GES-1, AGS, MKN-45, and MGC-803; Breast cell lines MCF-7, MCF-10A, and MDA-MB-231; Colon cell lines NCM460, HCT116, SW620, and RKO; Liver cell lines L-O2, HUH-7, HepG2, and SMMC-7721, were all incubated in RPMI-1640 supplemented with 10% FBS ( Hyclone) plus 1% antibiotics (100 U/mL penicillin and 100 µg/mL streptomycin) (MA0110, MEILUNE, China), and maintained at 37℃ in a 5% CO_2_ incubator (HF90-HT, Heal Force, China).

### Quantitative Real-time Polymerase Chain Reaction (qRT-PCR)

After the above-mentioned cells were pretreated, total RNA was extracted according to the instructions of the TRIzol reagent (Mei5bio, Wuhan, China). The absorbance values at 260 and 280 nm were measured by spectrophotometer to ensure that the RNA concentration and purity were consistent. RNA was reverse transcribed into cDNA according to the instructions of M5 Sprint qPCR RT kit with gDNA remover reverse transcription kit.

We used cDNA as a template and 2 × M5 HiPer SYBR Premix EsTaq (with Tli RnaseH) as a fluorescent dye for RT-qPCR detection. *CD274* primers were designed and synthesized by Wuhan Sevier Biotechnology company limited. The primer sequences are as follows: forward (5’-3’): AGAACTACCTCTGGCACAT-CCTC, reverse (5’-3’): AACGGAAGATGAATGTCAGTGCTA.

### Statistical analysis

To examine *CD274* expression in various tissues, the Kruskal–Wallis test and pair t-test were used. In survival analysis, univariate Cox regression analysis was used to get the HRs and *P* values. In order to compare the survival of patients who were expanded into groups based on the *CD274* expression levels, K-M curves were employed. *P* < 0.05 was considered statistically significant.

## Results

### Pan-cancers’ levels of *CD274* mRNA

**Fig. 1:**
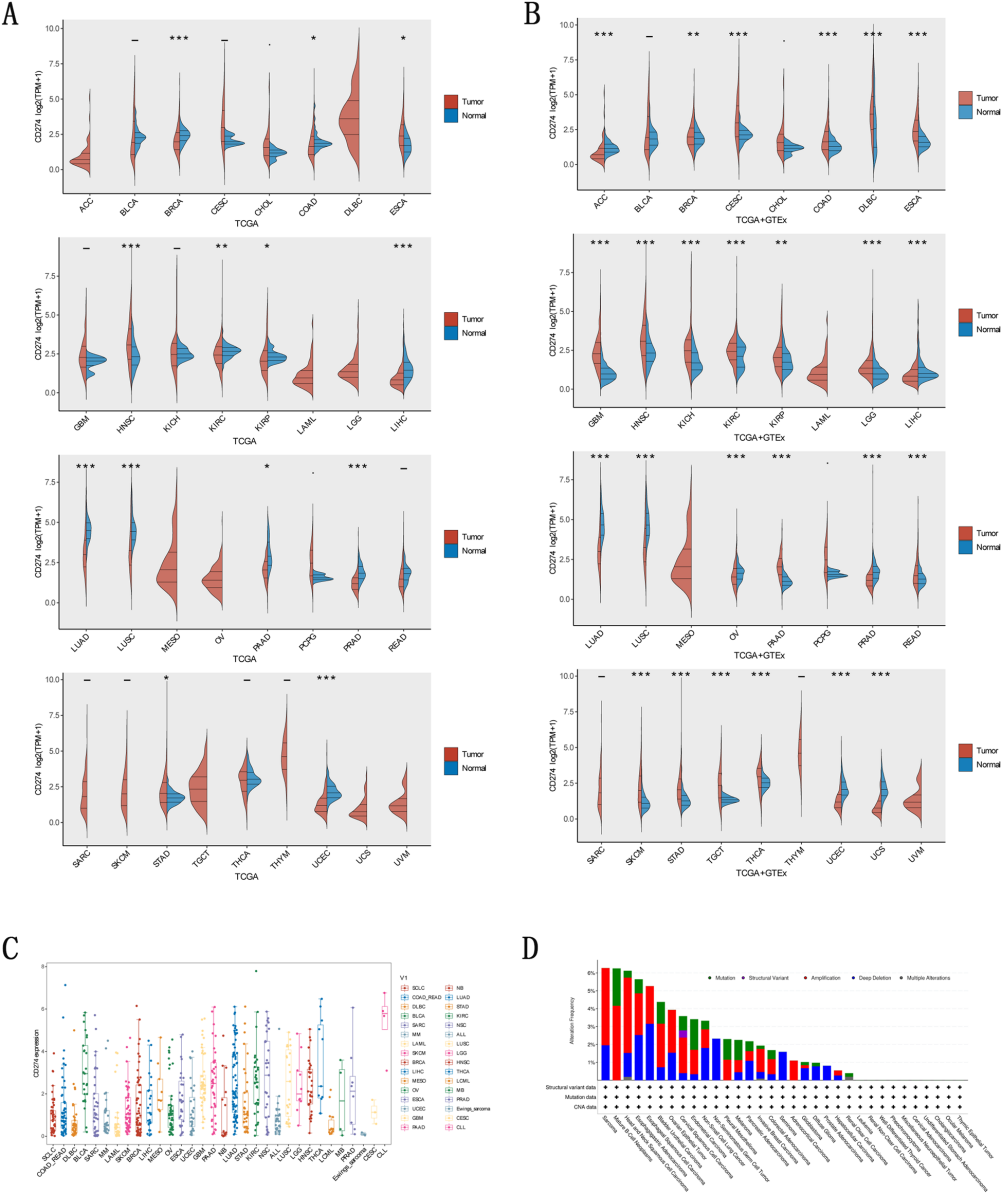
Aberrant expressions and genetic alterations of CD274 in pan-cancer. **A** Expression level of CD274 in tumor and normal tissues from TCGA database. **B** Expression level of CD274 in tumor and normal tissues from TCGA and the GTEx database. * *P* < 0.05;** *P* < 0.01; *** *P* < 0.001. **C** CD274 mRNA expression in different cancer types in TIMER. **D** The alteration frequency of CD274 in human pan-cancer

Using TCGA data, we assessed the levels of *CD274* expression in 33 cancer samples matched with normal samples additionally (Fig. 1A). In 13 forms of cancer, significant changes in *CD274* expression were found between tumor and normal tissue. *CD274* was found to be strongly expressed in HNSC (head and neck squamous cell carcinoma), STAD (stomach adenocarcinoma), and ESCA (esophageal carcinoma). In contrast, tumors have lower levels of *CD274* than normal tissues did in COAD (colon adenocarcinoma), KIRC (kidney renal clear cell carcinoma), UCEC (uterine corpus endometrial carcinoma), KIRP (kidney renal papillary cell carcinoma), PAAD (pancreatic adenocarcinoma), LUSC (lung squamous cell carcinoma), LUAD (lung adenocarcinoma), LIHC (liver hepatocellular carcinoma), PRAD (prostate adenocarcinoma), and BRCA (breast invasive carcinoma). Using the TCGA + GTEx data set, significant differences in *CD274* expression were found in 25 different tumors (Fig. 1B). Apart from the same data of 11 types of cancers presented by TCGA above, we also found that *CD274* was highly expressed in COAD, BRCA, DLBC (lymphoid neoplasm diffuse large B cell lymphoma), THCA (thyroid carcinoma), KIRP, LCG, CESC (cervical squamous cell carcinoma), TGCT (testicular germ cell tumors), PAAD, SKCM (skin cutaneous melanoma), and READ (rectum adenocarcinoma), downregulated in tumors relative to normal tissues in OV (ovarian serous cystadenocarcinoma), ACC (adrenocortical carcinoma), and UCS (uterine carcinosarcoma). After the t-test, we confirmed that significant differences existed in the expression of *CD274* between tumor and normal tissues in these cancers (Supplementary Fig. S1).

Then, based on CCLE data, we assessed *CD274* expression levels in a number of cell lines (Fig. 1C). All cancers expressed *CD274*, with Ewings-sarcoma expressing the least and CLL (Glioblastoma multiforme) expressing the most. To explore the reasons for *CD274*’s different expression in tumors, we identified genomic alterations of *CD274* in 33 cancers. We found that the highest gene alterations appear in SARC (sarcoma), with the main mutation type of amplification (Fig. 1D). What’s more, as shown in Supplementary Fig. S2A, the predominant mutation style in *CD274* was Missense. And Supplementary Fig. S2B presented the E188K/Vfs*18 mutation site in the three-dimensional protein structure of *CD274.* As for *CD274*’s putative copy-number alterations, the most frequent ones were amplification and gain function (Supplementary Fig. S2C).

### Prognostic value of *CD274* across cancers

**Fig. 2:**
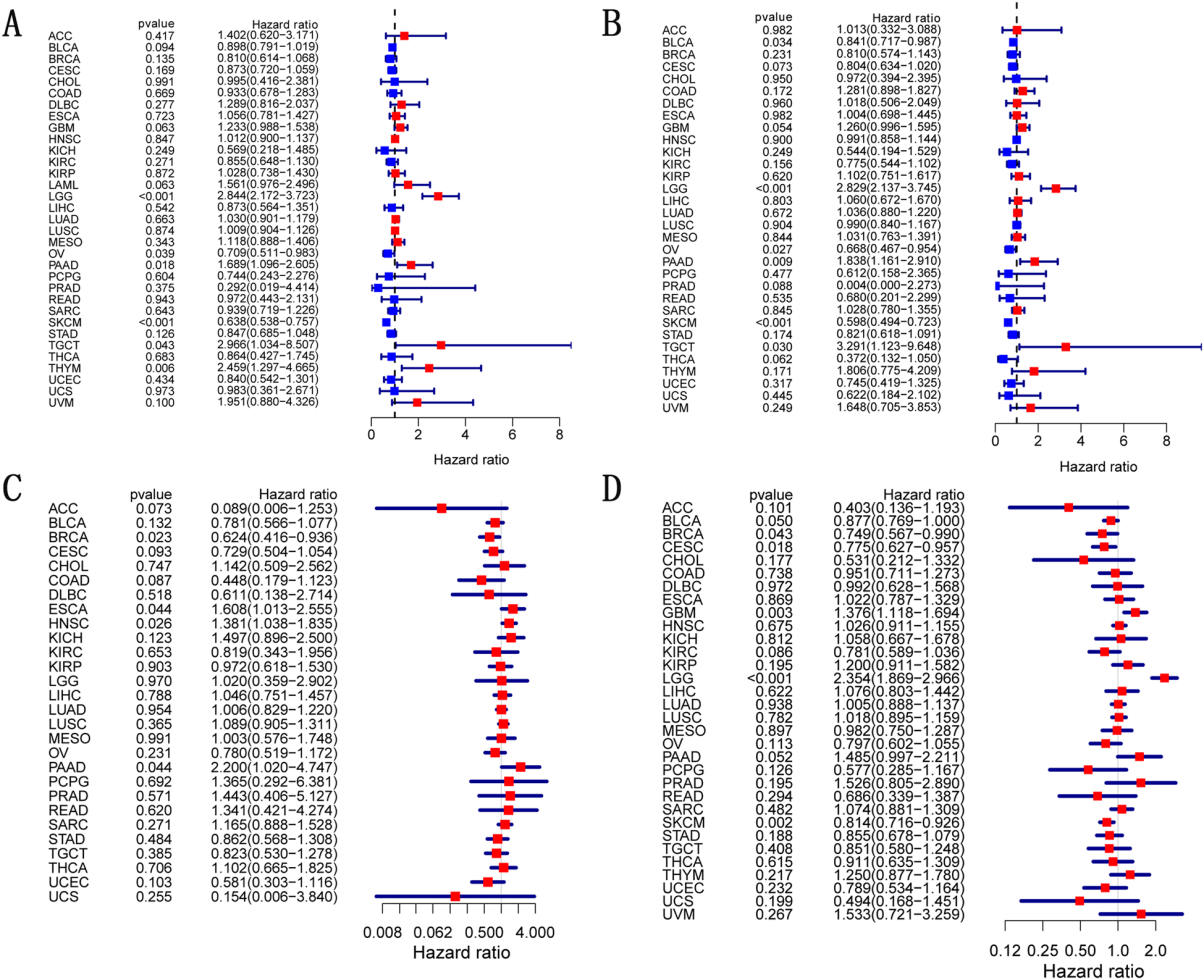
The forest maps of *CD274* expression level with survival in different types of cancers. Association between *CD274* expression level and patients’ OS (**A**), DSS (**B**), DFI (**C**), and PFI (**D**). Red squares represent the hazard ratio

For each malignancy, we performed a survival association study to present the association between *CD274* expression levels and prognosis. Notably, *CD274* expression was substantially connected with patients' overall survival in 6 distinct cancers, which were LGG (brain lower grade glioma), SKCM, THYM (thymoma), PAAD, OV, and TGCT (Fig. 2A). Besides, for K-M survival curves (OS), *CD274* expression is correlated with survival in patients with ACC, SKCM, LGG and THYM cancers, which suggests that *CD274* expression correlates with the prognosis of the four cancers (Supplementary Fig. S3A). (THYM, *P* = 0.017; SKCM, *P* < 0.001; ACC, *P* = 0.001; LGG, *P* < 0.001). *CD274* expression was found to influence patients' DFI (disease-free interval) in six cancer types, which were LGG, SKCM, PAAD, OV, TGCT, and BLCA(bladder urothelial carcinoma) (Fig. 2B). In particular, *CD274* played the role of a detrimental prognostic factor in PAAD, TGCT, and LGG. However, it was positively correlated with favorable outcomes in OV, BLCA, and SKCM (Fig. 2B). We looked examined the connection between *CD274* expression and DSS (disease-specific survival) in 33 malignancies because non-tumor-related variables could lead to death in the time of the follow-up. We could find that higher *CD274* expression was associated with a poor prognosis in HNSC, ESCA, and PAAD, but a good prognosis in BRCA (Fig. 2C). We also investigated the relationship between PFI (progression-free interval) and *CD274* expression. According to the results, increasing *CD274* expression in BLCA, BRCA, SKCM, and CESC negatively affects PFI but favorably affects GBM (glioblastoma multiforme) and LGG (Fig. 2D).

**Fig. 3:**
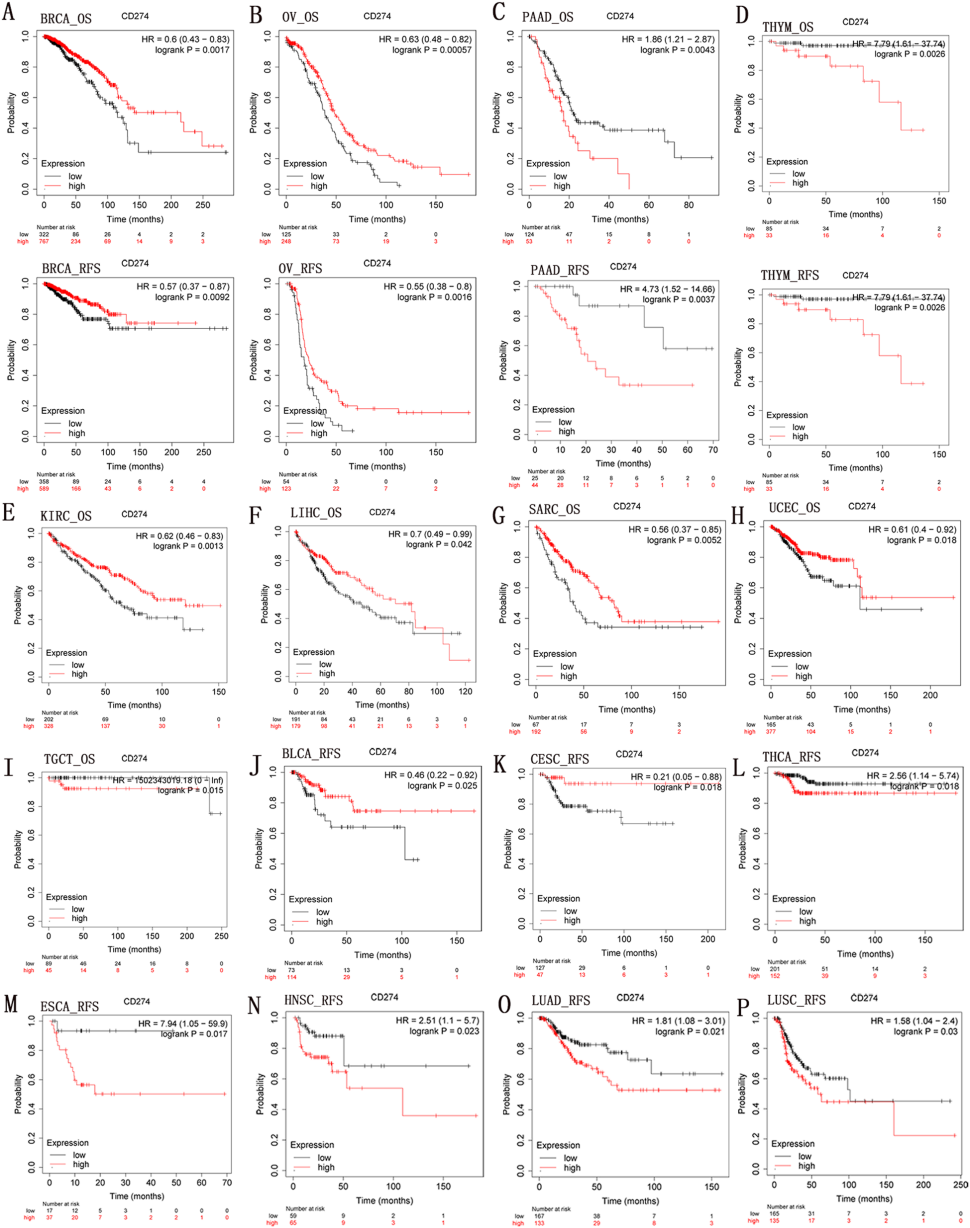
Kaplan-Meier survival curves comparing the high and low expression of* CD274* gene in various cancer types in Kaplan-Meier Plotter. OS and RFS of (**A**) BACA, (**B**) OV, (**C**) PAAD, and (**D**) THYM. OS of (**E)** KIRC, (**F**) LIHC, (**G**) SARC, (**H**) UCEC, and (**I**) TGCT. RFS of (**J**) BLCA, (**K**) CECS, (**L**) PCPG, (**M**) ESCA, (**N**) HNSC, (**O**) LUAD, (**P**) LUSC, and (**Q**) THCA

Then, we assessed *CD274*-related survival using a K-M plotter. As shown in Fig. 3, we can find that *CD274* played a predictive role for BRCA (OS: *P* = 0.0017), OV (OS: *P* = 0.00057), KIRC (OS: *P* = 0.0013), LIHC (OS: *P* = 0.042), SARC (OS: *P* = 0.0052), UCEC (OS: *P* = 0.018), BLCA, and CECS. While *CD274* expression had a detrimental effect in PAAD (OS: *P* = 0.0043), THYM (OS: *P* = 0.0026), TGCT (OS: *P* = 0.015), THCA, ESCA, HNSC, LUAD, and LUSC.

### Correlation of *CD274* expression with clinicopathology

**Fig. 4:**
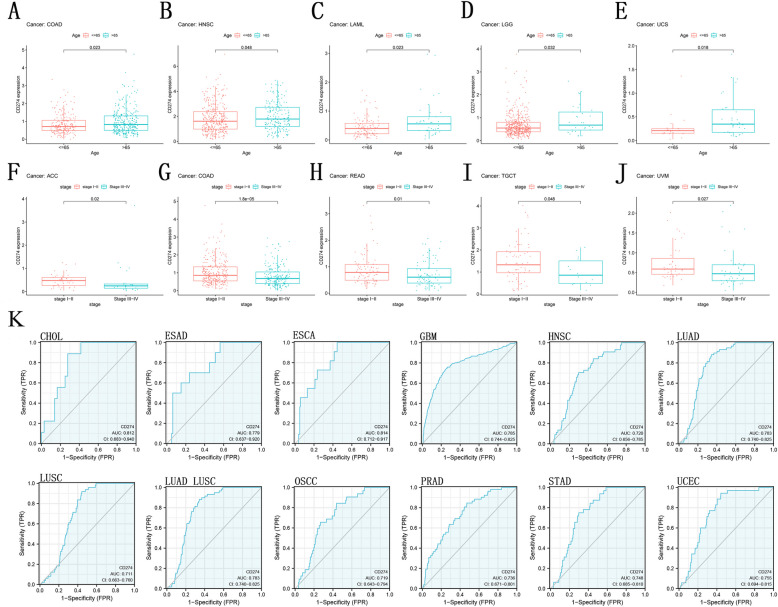
Association between the *CD274* gene expression and clinicopathological features in various cancer types. *CD274* gene expression relevant with age in COAD (**A**), HNSC (**B**), LAML (**C**), LGG (**D**), and UCS (**E**). CD274 gene expression related to the stage in ACC (**F**), COAD (**G**), READ (**H**), TGCT (**I**), and UVM (**J**). ROC curves of GBM, LUAD, LUADLUSC, CHOL, LUSC, PRAD, STAD, UCEC, ESCA, HNSC, OSCC, and ESAD in accordance with *CD274*-derived risk score (**K**)

Additionally, we looked at the age-related changes in *CD274* expression in patients suffering from each tumor type and discovered that older individuals had higher levels of expression in COAD, HNSC, LAML (acute myeloid leukemia), LGG, and UCS (Fig. 4A-E). Additionally, stage I–II patients with ACC, COAD, READ, TGCT, and UVM had higher levels of *CD274* expression than stage III–IV patients with those conditions (Fig. 4F-J). The *CD274* expression contributed the most to the prediction of OS duration. For GBM, LUAD, LUADLUSC, CHOL (cholangiocarcinoma), LUSC, PRAD, STAD, UCEC, ESCA, HNSC, OSCC, and ESAD (esophageal adenocarcinoma), all of them had an AUC above 0.7, which confirmed the satisfying efficiency in predicting patients’ survival outcomes (Fig. 4K). Moreover, for ESAD, GBMLGG (lower grade glioma and glioblastoma), LAML, LGG, PAAD, TGCT, and THYM, the vast majority of AUC values (1 year, 3 years, and 5 years) were greater than 0.6, which can predict a relatively high survival accuracy of 1, 3, and 5 years (Supplementary Fig. S4).

### TMB and MSI expression and *CD274* expression in pan-cancer

**Fig. 5:**
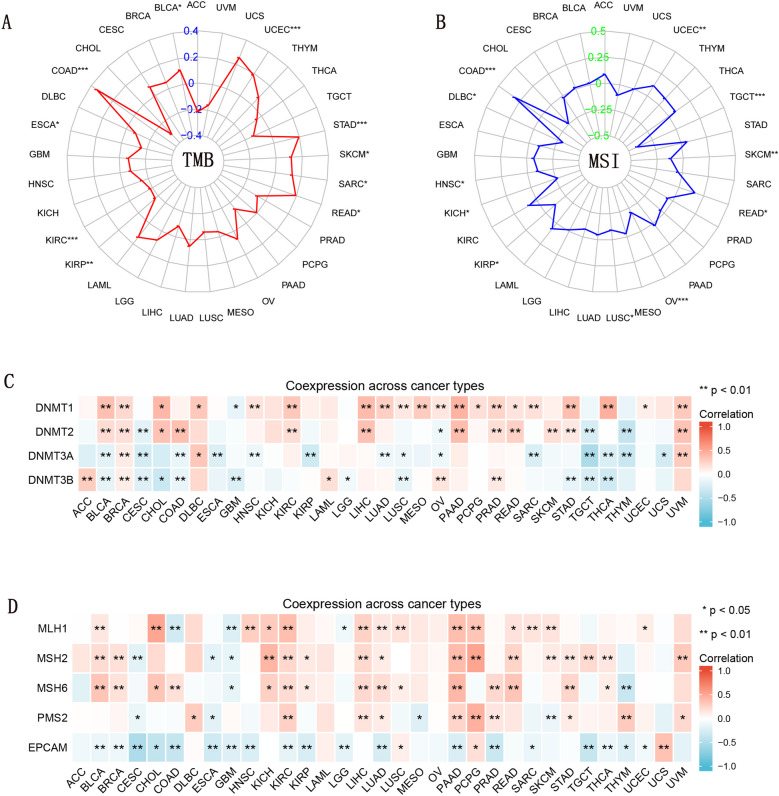
The correlation of *CD274* expression with immune-related biomarkers. **A** The radar chart showed the association between TMB and *CD274* gene expression in different cancers. The red curve denotes the correlation coefficient, and the blue value denotes the range. **B** The radar chart showed the relationship between MSI and *CD274* gene expression in different cancers. The blue curve represents the correlation coefficient, and the green value represents the range. **C** Heatmap indicating the correlation between *CD274* expression and DNMTs levels. **D** Heatmap indicating the correlation between *CD274* expression and MMR genes. For each pair, the top left triangle indicates the *p*-value, and the bottom right triangle indicates the correlation coefficient

High TMB is a novel and developing biomarker that correlates with immune checkpoint inhibitor sensitivity [32]. Our findings indicate a positive correlation between *CD274* expression and TMB in 7 malignancies, including UCEC, STAD, SKCM, SARC, READ, COAD, and BLCA. In 3 malignancies, including KIRP, KIRC, and ESCA, *CD274* expression, on the other hand, exhibited a negative connection with TMB (Fig. 5A).

Additionally, we looked into whether *CD274* expression might be associated with MSI in other malignancies. Results revealed that in 3 malignancies, *CD274* expression correlated well with MSI (UCEC, READ, and COAD). On the other hand, in 8 malignancies (TGCT, SKCM, OV, LUSC, KIRP, KICH (kidney chromophobe), HNSC, and DLBC), *CD274* expression demonstrated a negative connection with MSI (Fig. 5B).

In 22 of the 33 tumors, *CD274* expression correlated with the levels of at least two DNMTs, especially in BLCA, BRCA, and OV. DNMT1 and DNMT2 are positively correlated with most cancers, while DNMT3A and DNMT3B are inversely correlated with most cancers (Fig. 5C). What’s more, in 21 tumors out of 33 tumors, *CD274* expression is associated with the levels of at least three MMR-related genes, among which KIRC, LUAD, and PAAD show strong connections with all five genes. MLH1, MSH2, MSH6, and PMS2 are positively associated with most cancers, while EPCAM is inversely associated with most cancers (Fig. 5D).

### Correlation of Tumor Microenvironment (TME) and *CD274* expression

**Fig. 6:**
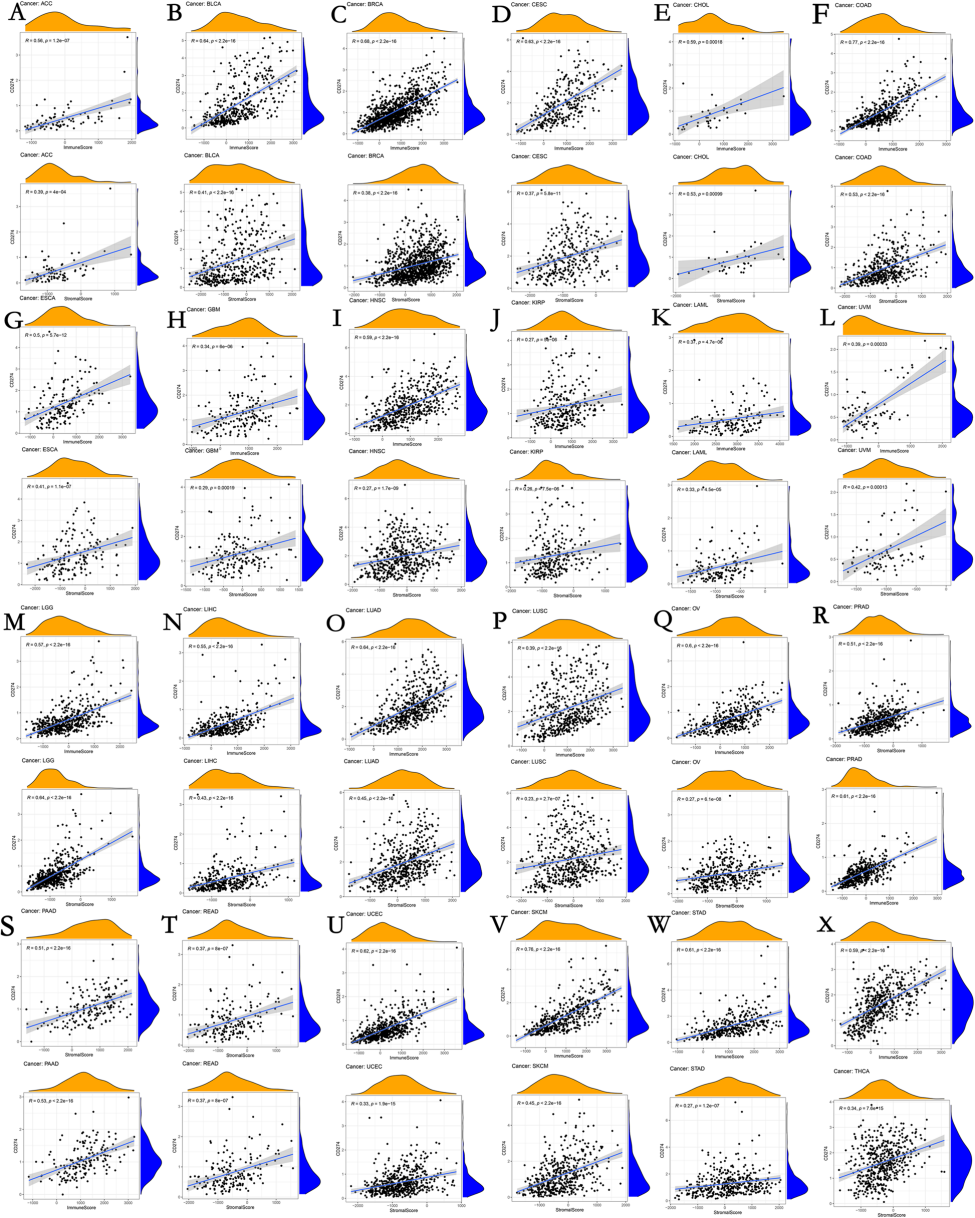
Correlation analysis between* CD274* expression and ImmuneScore/Stromal Score in human pan-cancer. **A** ACC. **B** BLCA. **C** BRCA. **D** CECS. **E** CHOL. **F** COAD. **G** ESCA. **H** GBM. **I** HNSC. **J** KIRP. **K** LAML. **L** UVM. **M** LGG. **N **LIHC. **O** LUAD. **P** LUSC. **Q** OV. **R** PRAD. **S** PRAD. **T** READ. **U** UCEC. **V** SKCM. **W** STAD. **X** THCA

The TME is crucial for promoting cancer cell heterogeneity, which raises drug resistance and facilitates the growth and metastasis of cancer cells [33]. Our findings revealed a substantial positive connection between *CD274* expression and stromal and immunological scores (Fig. 6) in SARC, UCEC, BLCA, THYM, BRCA, PCPG, CECS, LUAD, ESCA, OV, PAAD, HNSC, LIHC, KIRC, TGCT, LUSC, and THCA, showing that the quantity of stromal or immune cells rises concurrently with an increase in *CD274* expression levels.

### Analysis of TIICs

**Fig. 7:**
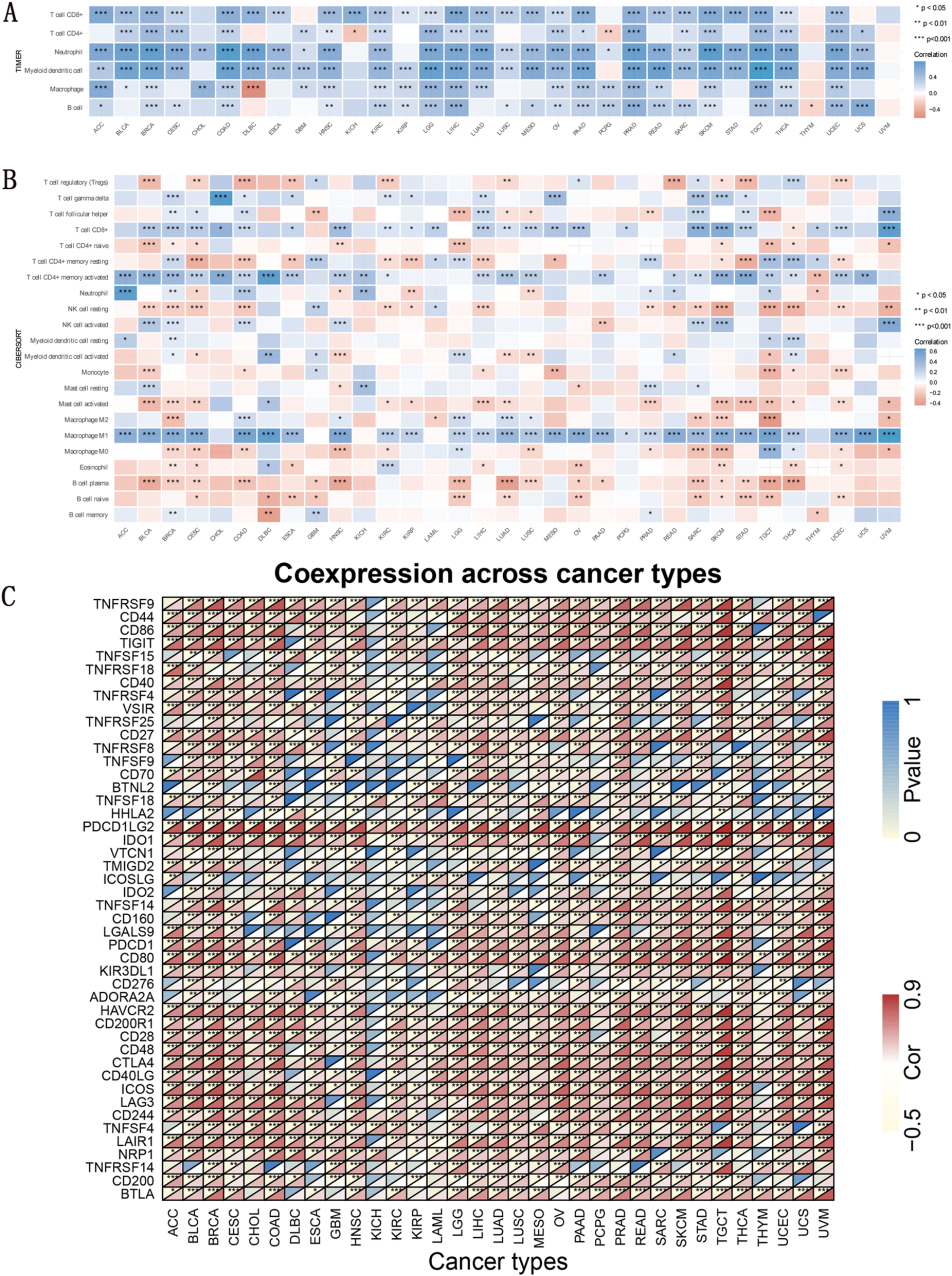
TIICs analysis and correlation analysis of *CD274* expression levels with immune checkpoints in human pancancer. **A** *CD274* expression and immune-associated cells infiltration in pan-cancer was further performed in the TIMER database. **B** *CD274* expression and the infiltrating levels of 22 immune-related cells using CIBERSORT algorithm. **C** The heatmap shows the correlation between *CD274* and immunosuppressive genes in TCGA pan-cancer. For each pair, the top left triangle indicates the *p*-value, and the bottom right triangle indicates the correlation coefficient

The TIMER database was used to investigate the connection between *CD274* expression and immune-associated cell infiltration in different tumors. In PRAD, LIHC, PAAD, COAD, LGG, HNSC, THCA, BRCA, KIRC, OV, SKCM, TGCT, and UCEC, we could find that the expression levels of *CD274* were strongly connected with six infiltrating immune-associated cells (Fig. 7A). So, using the CIBERSORT method, we looked at the connection between *CD274* expression and the numbers of 22 TIICs. Significant correlations were found between the amounts of TIICs and *CD274* expression. Our findings showed that the levels of some TIICs are substantially linked with *CD274* expression in BRCA (*n* = 18), CECS (*n* = 15), SKCM (*n* = 15), TGCT (*n* = 15), THCA (*n* = 14), COAD (*n* = 14), SARC (*n* = 13), HNSC (*n* = 11), BLCA (*n* = 11), and UCEC (*n* = 11) (Fig. 7B). We further chose 47 immunosuppressive marker genes and conducted a *CD274* correlation analysis. *CD274* is positively correlated with *PDCD1LG2, TNFRSF9*, *CD80*, *HAVCR2*, *CD200R1*, *ICOS*, *TIGIT*, and *CTLA4,* while is negatively correlated with *BTNL2* and *VTCN1* (Fig. 7C) in many tumor types in these immunosuppressive marker genes.

### *CD274* expression level in immune subtypes

Additionally, we obtained *CD274* expression data from the TISDB website (http://cis.hku.hk/TISIDB/index.php) for use in analyzing the relationship between the immune system and tumor molecular subtypes. The findings showed a strong correlation between *CD274* expression and immune subtype including C1-C6 of PRAD, SKCM, UVM, SARC, MESO, LIHC, BLCA, OV, LUAD, BRCA, LGG, CECS, UCS, COAD, HNSC, LUSC, PCPG (pheochromocytoma and paraganglioma), STAD, READ, UCEC, and TGCT (uveal melanoma) (Supplementary Fig. S5). While the expression of *CD274* in the immune subtype of GBM, KICH, ACC, THCA, ESCA, KIRC, CHOL, KIRP, and PAAD was not statistically different (data not presented). The findings revealed a strong correlation between *CD274* expression and the molecular subtype of PRAD, COAD, BRCA, HNSC, ACC, KIRP, UCEC, LGG, STAD, OV, PCPG, READ, and LUSC (Supplementary Fig. S6).

### *CD274* drug sensitivity analysis

**Fig. 8:**
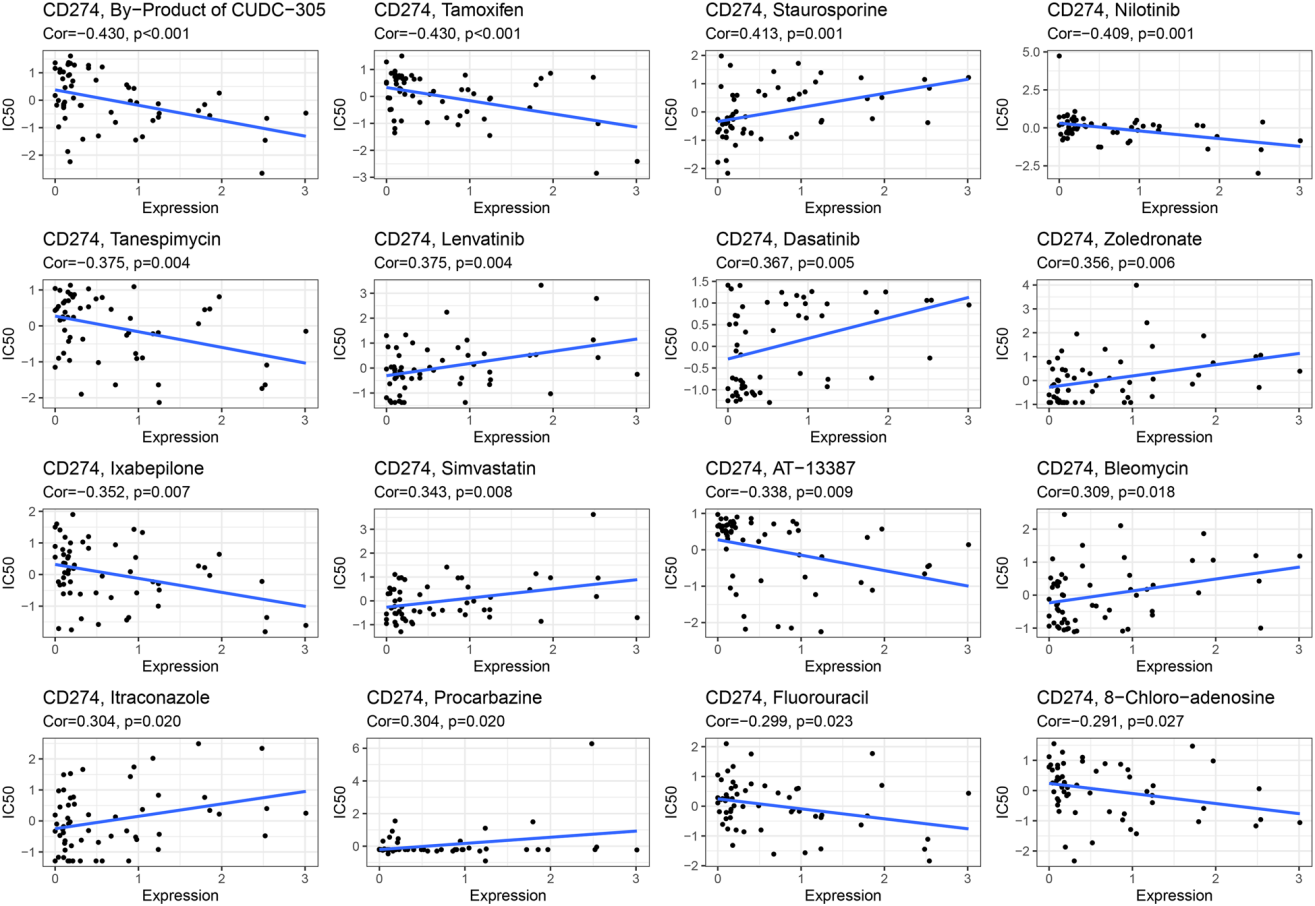
Drug sensitivity analysis of *CD274*. The expression of *CD274* was correlated with the sensitivity of the By−Product of CUDC−305, Tamoxifen, Staurosporine, Nilotinib, Tanespimycin, Lenvatinib, Dasatinib, Zoledronate, Ixabepilone, Simvastatin, AT−13387, Bleomycin, Itraconazole, Procarbazine, Fluorouracil, and 8−Chloro−adenosine

Using the CellMinerTM database (https://discover.nci.nih.gov/cellminer/home.do), we looked at the possibility of a link between drug sensitivity and *CD274* expression (Fig. 8). Notably, the drug sensitivity of CUDC-305’s byproducts, tamoxifen, nilotinib, tanespimycin, ixabepilone, AT-13387, fluorouracil, and 8-chloro-adenosine was adversely linked with *CD274* expression. Our findings showed that staurosporine, lenvatinib, dasatinib, zoledronate, simvastatin, bleomycin, itraconazole, and procarbazine were positively correlated with *CD274* expression.

### *CD274*’s biological role in cancer

**Fig. 9:**
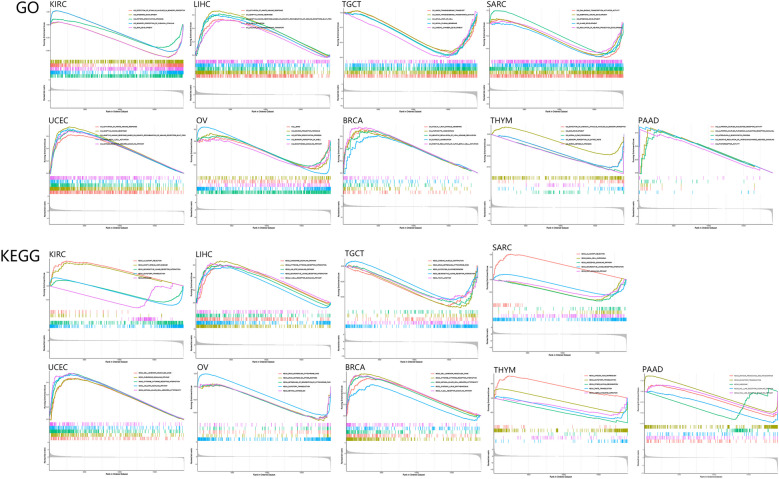
Pathway analysis of *CD274* in different cancers. **A** GO functional annotation of *CD274* gene in a variety of tumors. **B** KEGG pathway analysis of *CD274* gene in a variety of tumors. Different color curves represent different functions or pathways. The positive and negative regulation of *CD274* is expressed as the peaks of the rising and falling curves respectively

GSEA was applied to study the primary biological function of *CD274* in tumors. As we know, GO functional enrichment analysis can indicate the differentially expressed genes are mainly enriched in different pathways, like chemokine regulation, angiogenesis regulation, and so on [34]. And in the context of GO functional annotation, *CD274* is associated with signaling pathways in KIRC, OV, SARC, TGCT, THYM, BRCA, LIHC, PAAD, and UCEC (Fig. 9A). Among them, lymphocyte homeostasis, activation of the innate immune response, antigen receptor-mediated signaling pathway, and platelet aggregation are linked closely to immunity or cancer and they are up-regulated pathways. Data from the examination of KEGG gene sets revealed that *CD274* influenced signaling pathways in KIRC, OV, SARC, TGCT, THYM, BRCA, LIHC, PAAD, and UCEC (Fig. 9B). Additionally, we discovered that the control of *CD274* was complicated in the tumors KIRC, SARC, THYM, and PAAD (Fig. 9B). T-cell receptor signaling, NKT cell-mediated cytotoxicity, and JAK-STAT signaling are up-regulated pathways, while toll-like receptor signaling and Wnt signaling are down-regulated pathways. In the meantime, these five pathways are associated with immunity or cancer. Furthermore, by presenting the interaction network of cells, microRNAs, and secreted factors associated with *CD274* in the TME, we found that some basic signaling pathways play significant regulatory roles, such as PI3K signaling and Ras signaling (Supplementary Fig. S7). As indicated above, these pathways and functions are basically tumor related.

### *CD274*’s protein expression and mRNA expression in different cancers

**Fig. 10:**
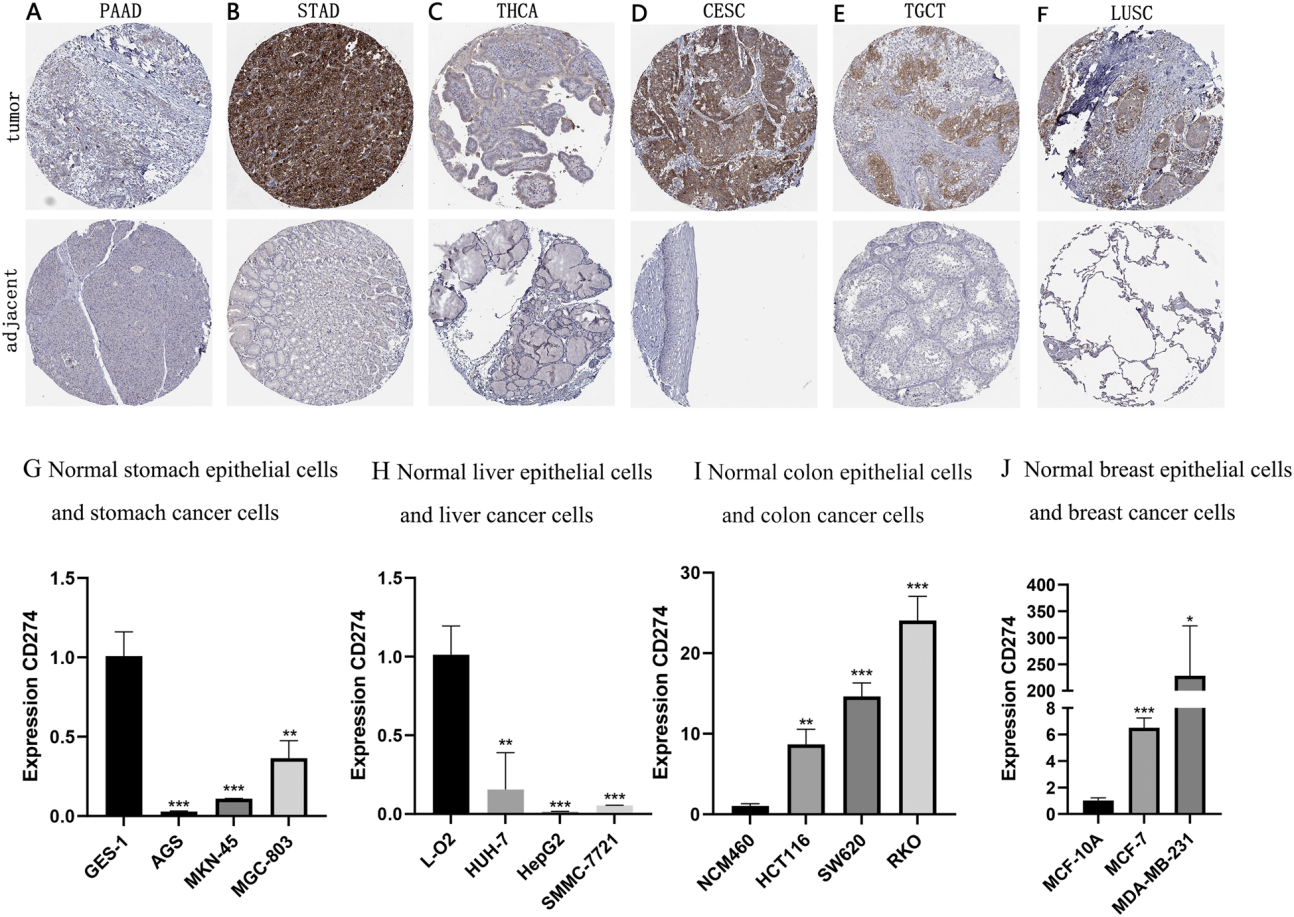
*CD274*’s protein expression levels in tumors of PAAD (**A**), STAD (**B**), THCA (**C**), CECS (**D**), TGCT (**E**), and LUSC (**F**). The mRNA expression of *CD274* in gastric cell lines GES-1, AGS, MKN-45, and MGC-803 (**G**); The mRNA expression of *CD274* in liver cell lines L-O2, HUH-7, HepG2, and SMMC-7721 (**H**); The mRNA expression of *CD274* in colon cell lines NCM460, HCT116, SW620, and RKO (**I**); The mRNA expression of *CD274* in breast cell lines MCF-10A, MCF-7, and MDAMB-231 (**J**). * *P*＜0.05, ** *P*＜0.01, and *** *P*＜0.001

Moreover, the HPA database was used to evaluate the expression of *CD274* among different tumors. From the data, we can see that higher *CD274* expression levels were observed in different tumors (PAAD, STAD, THCA, CECS, TGCT, and LUSC) (Fig. 10A-F). Among them, the *CD274*’s protein expressions of PAAD, STAD, THCA, CECS, and TGCT were consistent with the bioinformatics analysis. Furthermore, the results of PCR showed that the expressions of *CD274* in stomach cancer cells (AGS, MKN-45, and MGC-803) and liver cancer cells (HUH-7, HepG2, and SMMC-7721) were significantly lower than that in their normal cells (Fig. 10G and H). *CD274* expressions in colon cancer cells HCT116, SW620, and RKO were higher compared with normal cells (Fig. 10I). Furthermore, *CD274* was highly expressed in breast cancer cells MCF-7 and MDA-MB-231 compared to normal cells (Fig. 10J). Based on the results, we can infer that *CD274* may be of significance in various cancers.

### Relationship between *CD274* expression and prognosis of LGG and SKCM

**Fig. 11:**
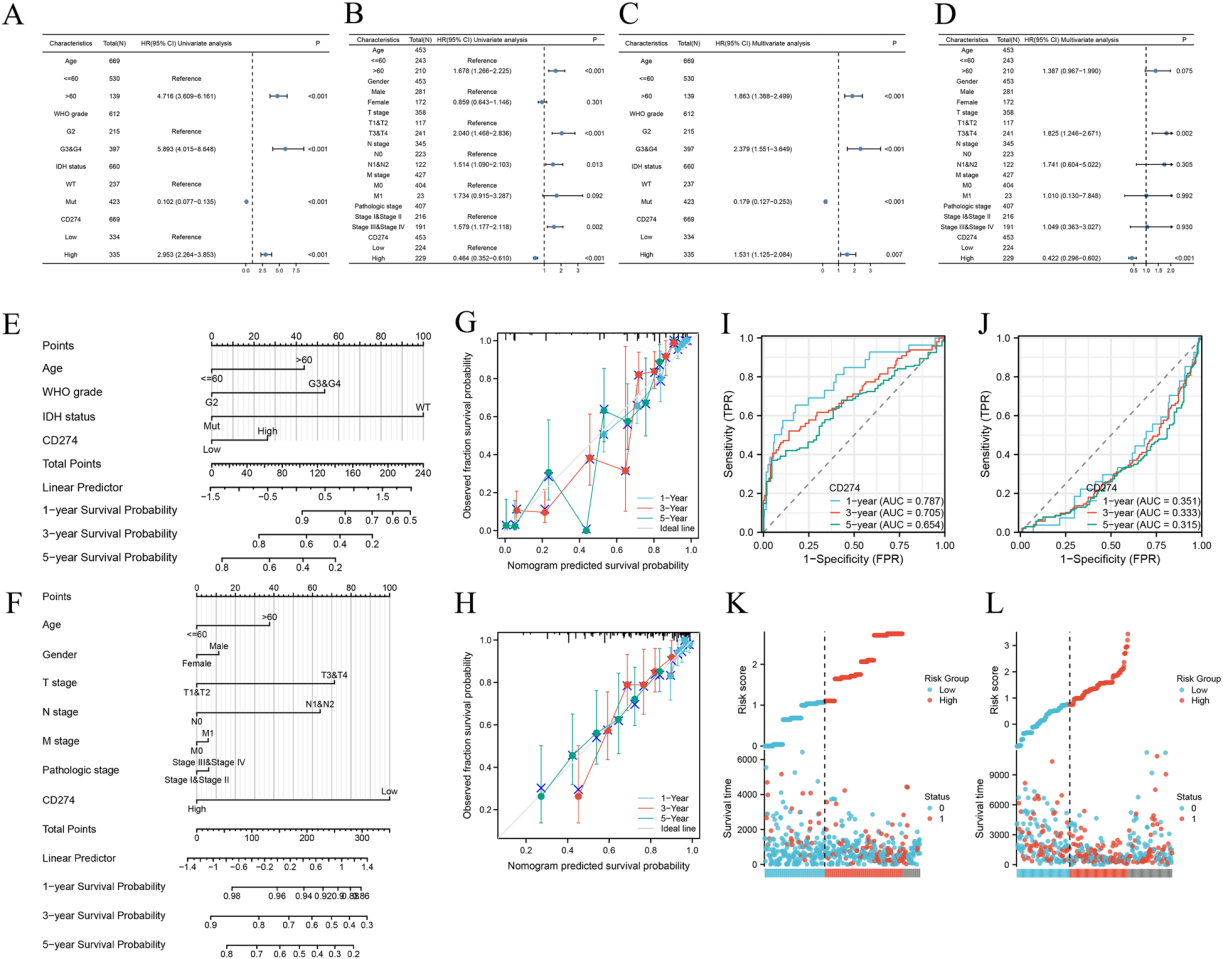
Relationship between *CD274* expression and prognosis of LGG and SKCM. Univariate Cox regression analysis of OS in LGG (**A**) and SKCM (**B**). Multivariate Cox regression analysis of OS in LGG (**C**) and SKCM (**D**). Nomogram for 1-year, 3-year and 5-year OS of LGG (**E**) and SKCM (**F**) patients. Calibration plots for 1-year, 3-year and 5-year OS prediction of LGG (**G**) and SKCM (**H**). Time-dependent ROC curves and AUC values for 1-year, 3-year and 5-year OS prediction of LGG (**I**) and SKCM (**J**). *CD274* expression, risk score and survival time distribution of LGG (**K**) and SKCM (**L**)

To further analyze the relationship between *CD274* and disease prognosis, we conducted further studies specifically on LGG and SKCM. As for LGG, from the univariate analysis, the results indicated that age, WHO grade, IDH1 status, and *CD274* expression level were significantly associated with the OS (*P* < 0.001 for all, Fig. 11A). As for SKCM’s univariate analysis, age, gender, TNM stage, pathological stage, and *CD274* expression level were also significantly associated with the OS (*P* < 0.05 for most, Fig. 11B). These risk factors for LGG and SKCM were further included in multivariate Cox regression, which suggested that *CD274* was an independent prognostic factor (LGG:HR = 1.531, 95%CI = 1.125–2.084, *P* = 0.007, Fig. 11C; SKCM:HR = 0.422, 95%CI = 0.296–0.602, *P* < 0.001, Fig. 11D). Clinical characteristics were incorporated into the nomogram model (Fig. 11E and F). We then developed time-dependent ROC curves and calibration plots predicting the probability of 1-year, 3-year, and 5-year OS rates (Fig. 11I and J). The AUCs of LGG (1-year, 3-year, and 5-year) were 0.787, 0.705, and 0.654, while SKCM’s AUCs were 0.351, 0.333, and 0.315, respectively. The predicted probabilities of the calibration plots were consistent with the observed results (Fig. 11G and H). Furthermore, correlations between risk scores, survival times, and *CD274* expression profiles were subsequently investigated and demonstrated (Fig. 11K and L).

## Discussion

Pan-cancer analysis, a study of molecular abnormalities data from several cancer kinds [35], can reveal commonalities and differences between tumors, giving insight into the design of diagnostic targets [1, 36]. In previous studies, researchers developed various prognostic prediction models. For instance, *AC010973.2*, one of six stemness-related genes, can promote cell proliferation and predict overall survival in renal clear cell carcinoma [37]. Besides, the role of autophagy-related genes in COAD was revealed to facilitate the design of new targets for improving cancer therapy [1]. Apart from these, pan-cancer analysis can spot patterns in critical biological functions that are dysregulated in cancer cells of various ancestries [38]. The B7 family of immune-regulatory molecules includes *PD-L1*, also referred to as *B7-H1* or *PD-L1*, which is an immunological co-signaling molecule [39]. However, pan-cancer analysis of *CD274* regulation in human pan-cancer has not yet been clarified [40].

In the current study, we discovered that *PD-L1* is abnormally expressed in 25 different cancer types and its levels and DNA methylation are highly linked with MMR gene mutation levels. Additionally, patients’ prognoses, particularly those with LGG, SKCM, THYM, PAAD, OV, TGCT, BRCA, KIRC, LIHC, SARC, and UCEC, were linked to *PD-L1* expression. The expression of immune checkpoint markers and immune infiltration levels were also found to be favorably linked with *PD-L1* expression, particularly in BRCA, COAD, HNSC, SKCM, TGCT, THCA, and UCEC. These findings clearly suggest that *PD-L1* may be a predictive biomarker for those malignancies.

The DNA damage repair mechanism known as MMRs is made up of several heterodimers. The accumulation of DNA replication mistakes caused by the functional loss of important genes in this pathway increases somatic mutation rates, MSI, and cancer [41, 42]. The previous study showed poor prognosis in prostate cancer patients and their sensitivity to Olaparib was closely related to mutations in the DNA damage response pathway [43]. While in our correlation analysis, we discovered in this study that *PD-L1* expression was strongly correlated with the mutation levels of 5 MMR genes in human pan-cancer. Changes in DNA methylation status also play a role in the growth of cancer. According to research, cancer frequently exhibits hypermethylation of the gene promoter [44, 45]. Additionally, we found a significant link between *PD-L1* expression and four DNMTs, particularly in the cases of BRCA, OV, UVM, and BLCA. The results above support the hypothesis that aberrant *PD-L1* expression might significantly influence carcinogenesis by modulating DNA methylation and MMR gene mutation levels.

TME is made up primarily of the extracellular matrix (ECM), the vasculature, and other benign cells that surround the tumor. It also contains elements that either encourage or prevent the growth of tumors, such as immune cells that are present in and around the tumor but are not carcinogenic (B cells, TILs, and T cells) [32, 46]. These non-cancerous elements have been demonstrated to play a crucial function in tumors as a double-edged sword to promote or inhibit tumor progression. They also significantly affect tumor sample genomic analyses and may alter how the data are biologically interpreted. *PD-1* is a crucial immunosuppressive transmembrane protein expressed on the surface of T cells. In the tumor microenvironment, tumor cells can express the ligand of *PD-1*, namely *PD-L1* or *PD-L2*. A well-known method for cancer cells to avoid T cell surveillance is that *PD-L1* binds to the *PD-1* of T cells [47, 48]. An essential mechanism for preserving immunological tolerance and preventing autoimmune disorders is the *PD-1*/*PD-L1* axis [49]. The balance between tumor immune surveillance and immunological resistance is also influenced by the *PD-1*/*PD-L1* axis. T cells become exhausted as a result of increased *PD-L1* expression on a tumor cell or TIL, reducing tumor-specific immunity and accelerating tumor growth [50]. Additionally, tumor-infiltrating T cells and tumor cells compete with one another. High quantities of aerobic glycolysis are seen in the first. Additionally, studies have demonstrated that highly glycolytic tumor cells are probable to deplete the microenvironment of glucose and other nutrients, which is essential for tumor-infiltrating T cells. So infiltrating T cells’ ability to respond to the tumor cells is dampened [50, 51]. Additionally, *PD-L1* has the ability to prevent activation of the RAS-ERK1/2 signaling pathway, which in turn prevents the proliferation of T lymphocytes, inhibits the activation of PKCδ, and lowers the level of IL-2 secreted by T cells [48, 52]. In the tumor microenvironment, some studies have confirmed that *PD-L1* is highly expressed in tumor cells as well as immune cells (Tregs, DCs, macrophages, and so on) [53]. Also, some studies have found *PD-L1* is known to be upregulated in response to interferon-γ produced by infiltrating T cells [54]. In our study, for most tumors, it was found that *PD-L1* expression had a strong positive correlation with T cell CD8 + and T cell CD4 + , and macrophages, which is consistent with the results of previous studies. It was found in previous studies that late GC B cells upregulate *PD-L1* [55]. Also, with high levels of *PD-L1* expression, humoral reactions can be significantly inhibited by regulatory B (Breg) cells [56]. For instance, B cells in terminal differentiation toward antibody secretors, in the presence of certain antigens, can be transcriptionally reprogrammed to produce high levels of inhibitory molecules, such as *PD-L1*, which suppress pro-inflammatory populations in the bone marrow and lymph [57]. In our study, for most tumors, it was found that *PD-L1* expression was strongly positively correlated with B cells, which is consistent with the results of the above studies.

Apart from wide expressions on the surface of macrophages, B lymphocytes, DCs, and T lymphocytes, the surface of many tumor cells also presents high *PD-L1* expression, which leads to T cell exhaustion and immune tolerance, causing immune escape [21]. To sum up, the expression of *PD-L1* can be induced when a variety of cytokines and exosomes exist in the TME, which contributes to strengthening the *PD-L1*/*PD-1* signal to inhibit CTL activation in the TME and thereby boost tumor escape [44, 58].

However, there is not enough research on *PD-L1*'s functions in the immunological microenvironment. In this investigation, we discovered a significant association between *PD-L1* expression and immune cells that were infiltrating OV, BRCA, UCEC, COAD, LGG, KIRC, LIHC, PAAD, SKCM, TGCT, THCA, PRAD, and HNSC, which indicates that *PD-L1* may lead to the inhibition of tumor or tumorigenesis by altering the TIL status. The innovative researches represent a significant advancement in understanding *PD-L1*’s critical function in immune infiltration.

We use immunological scoring to gauge the quantity of invading CD3 + /CD45RO + , CD3 + /CD8 + , or CD8 + /CD45RO + lymphocytes at the tumor's center and borders. A higher ImmuneScore or StromalScore represents that the TME has more immune or matrix components [59]. Our findings showed a substantial positive connection between *PD-L1* expression and stromal and immunological scores in these malignancies, showing that the quantity of stromal or immune cells rises concurrently with an increase in *PD-L1* expression levels. Additionally, we can see that immunological scores in the DLBC, KIRC, MESO, SARC, TGCT, and USC have a favorable connection with *PD-L1* expression. Additionally, the relationship between immunological check-point markers and *PD-L1* expression suggests that *PD-L1* has a function in controlling tumor immunology in malignancies, particularly in BRCA, PRAD, LUAD, BLCA, and OV. These findings further support the crucial part *PD-L1* plays in tumor immunity.

## Conclusion

By combining bioinformatic analysis and laboratory experiments, the characteristics of *PD-L1* were presented in a systematic manner by the pan-cancer study in a number of areas, such as expression pattern, genetic mutation, survival prognosis, MSI, TMB, MMR, tumor immune micro milieu, medication sensitivity, and signaling pathway. Since *PD-L1* showed abnormal expression in a number of cancers and predicted prognosis in patients suffering from these cancers, particularly for those with tumors like LGG, SKCM, THYM, PAAD, OV, TGCT, BRCA, KIRC, LIHC, SARC, and UCEC, it provides guidance for better strategies for the clinical treatment of immune checkpoint inhibitors. Furthermore, across a variety of cancer types, the aberrant *PD-L1* expression was connected to the MSI, MMR, TMB, and TIME. This work shows the several functions of *PD-L1* in pan-cancer and offers fresh information on *PD-L1*'s potential role in controlling chemoresistance. However, we do not have enough experiments to support this. Integrated meta-analysis in combination with existing studies and larger samples are needed to validate the role and mechanism of *PD-L1* in pan-cancer.

### Supplementary Information

**Additional file 1: Supplementary Fig. S1.** T test of *CD274* differential expression between tumor tissues and normal tissues in LUSC, HNSC, UCEC, PRAD, BLCA, CHOL, BRCA, ESCA, KICH, KIRP, LUAD, STAD, PRAD, READ, THCA, LIHC, COAD, and KIRC. * *P* < 0.05; ** *P* < 0.01; *** *P* < 0.001. **Supplementary Fig. S2.** Mutation of *CD274.* The site, type, and number of mutations in the *CD274 *gene (A). The E188K/Vfs*18 mutation site in the three-dimensional protein structure of *CD274 *(B). *CD274*’s alteration types in pan-cancer (C). **Supplementary Fig. S3.** Correlation between *CD274* expression level and patients’ survival level. K-M survival curves for ACC, LGG, SKCM, and THYM’s OS (A), BRCA, COAD, ESCA, and HNSC’s DSS (B), ACC, LGG, OV, SKCM, THYM, and UCS’s DFI (C), and ACC, CHOL, GBM, LGG, SKCM, and PAAD’s PFI (D). **Supplementary Fig. S4.** ROC curves (1-Year, 3-Year, and 5-Year) of ESAD, GBMLGG, LAML, LGG, PAAD, TGCT, and THYM in accordance with *CD274*-derived risk score. **Supplementary Fig. S5.** *CD274* expression in immune subtypes in LUAD, BLCA, CECS, BRCA, COAD, HNSC, LIHC, LUSC, MESO, PCPG, PRAD, OV, READ, SARC, SKCM, STAD, LGG, TGCT, UCS, UCEC, and UVM based on TISDB. **Supplementary Fig. S6.** *CD274* expression in a variety of molecular subtypes of OV, HNSC, ACC, COAD, BRCA, KIRP, LUSC, PCPG, PRAD, READ, STAD LGG, and UCEC by TISDB analysis. **Supplementary Fig. S7.** Significant biological pathways related to *CD274*. Cancer immunotherapy by *CD274* blockade (A). Interaction of immune cells and microRNAs in the tumor microenvironment (B).

## Data Availability

The raw data of this study are freely available from the website TCGA ResearchNetwork(https://portal.gdc.cancer.gov/), GTEx(http://commonfund.nih.gov/GTEx/), CCLE database(https://portals.broadinstitute.org/ccle/), TIMER database(https://cistrome.shinyapps.io/timer/), Kaplan Meier plotter portal(https://kmplot.com/analysis/), cBioPortal database (http://cbioportal.org), and HPA(https://www.proteinatlas.org/). All the analyzed data are included in the manuscript (and its supplementary information files). In addition, the supplementary materials can be found online.

## Abbreviations

PD-1: Programmed death-1
PD-L1(CD274): Programmed cell death 1 ligand 1
TCGA: The Cancer Genome Atlas
GTEx: Genotypic Tissue Expression Project
TMB: Tumor mutation burden
MSI: Microsatellite instability
MMR: Mismatch repair
DNMTs: DNA methyltransferases
GO: Gene Ontology
KEGG: Kyoto Encyclopedia of Genes and Genomes
TIGIT: T cell immune receptor with Ig and ITIM domains
CTLA4: Cytotoxic T Iymphocyte associate protein-4
OS: Overall survival
DFS: Disease-free survival
ACC: Adrenocortical Carcinoma
BLCA: Bladder Urothelial Carcinoma
BRCA: Breast invasive carcinoma
CESC: Cervical squamous cell carcinoma and endocervical adenocarcinoma
CHOL: Cholangiocarcinoma
COAD: Colon adenocarcinoma
DLBC: Lymphoid Neoplasm Diffuse Large B-cell Lymphoma
ESCA: Esophageal carcinoma
GBM: Glioblastoma multiforme
HNSC: Head and Neck squamous cell carcinoma
KICH: Kidney Chromophobe
KIRC: Kidney renal clear cell carcinoma
KIRP: Kidney renal papillary cell carcinoma
LAML: Acute Myeloid Leukemia
LGG: Lower Grade Glioma
LIHC: Liver hepatocellular carcinoma
LUAD: Lung adenocarcinoma
LUSC: Lung squamous cell carcinoma
MESO: Mesothelioma
OV: Ovarian serous cystadenocarcinoma
PAAD: Pancreatic adenocarcinoma
PCPG: Pheochromocytoma and Paraganglioma
PRAD: Prostate adenocarcinoma
READ: Rectum adenocarcinoma
SARC: Sarcoma
SKCM: Skin Cutaneous Melanoma
STAD: Stomach adenocarcinoma
TGCT: Testicular Germ Cell Tumors
THCA: Thyroid carcinoma
THYM: Thymoma
UCEC: Uterine Corpus Endometrial Carcinoma
UCS: Uterine Carcinosarcoma
UVM: Uveal Melanoma
COAD/READ: Colon and rectal cancer
