# Supplementary material for: Prognostic value and immunological role of PD-L1 gene in pan-cancer
Source: BMC Cancer. 2024 Jan 2;24:20. doi: 10.1186/s12885-023-11267-6 (PMC10763229; doi:10.1186/s12885-023-11267-6)
Supplement: Supplementary file 1 — Additional file 1: Supplementary Fig. S1. T test of CD274 differential expression between tumor tissues and normal tissues in LUSC, HNSC, UCEC, PRAD, BLCA, CHOL, BRCA, ESCA, KICH, KIRP, LUAD, STAD, PRAD, READ, THCA, LIHC, COAD, and KIRC. * P < 0.05; ** P < 0.01; *** P < 0.001. Supplementary Fig. S2. Mutation of CD274. The site, type, and number of mutations in the CD274 gene (A). The E188K/Vfs*18 mutation site in the three-dimensional protein structure of CD274 (B). CD274’s alteration types in pan-cancer (C). Supplementary Fig. S3. Correlation between CD274 expression level and patients’ survival level. K-M survival curves for ACC, LGG, SKCM, and THYM’s OS (A), BRCA, COAD, ESCA, and HNSC’s DSS (B), ACC, LGG, OV, SKCM, THYM, and UCS’s DFI (C), and ACC, CHOL, GBM, LGG, SKCM, and PAAD’s PFI (D). Supplementary Fig. S4. ROC curves (1-Year, 3-Year, and 5-Year) of ESAD, GBMLGG, LAML, LGG, PAAD, TGCT, and THYM in accordance with CD274-derived risk score. Supplementary Fig. S5. CD274 expression in immune subtypes in LUAD, BLCA, CECS, BRCA, COAD, HNSC, LIHC, LUSC, MESO, PCPG, PRAD, OV, READ, SARC, SKCM, STAD, LGG, TGCT, UCS, UCEC, and UVM based on TISDB. Supplementary Fig. S6. CD274 expression in a variety of molecular subtypes of OV, HNSC, ACC, COAD, BRCA, KIRP, LUSC, PCPG, PRAD, READ, STAD LGG, and UCEC by TISDB analysis. Supplementary Fig. S7. Significant biological pathways related to CD274. Cancer immunotherapy by CD274 blockade (A). Interaction of immune cells and microRNAs in the tumor microenvironment (B). [file 12885_2023_11267_MOESM1_ESM.docx]

**Supplementary Materials**

**
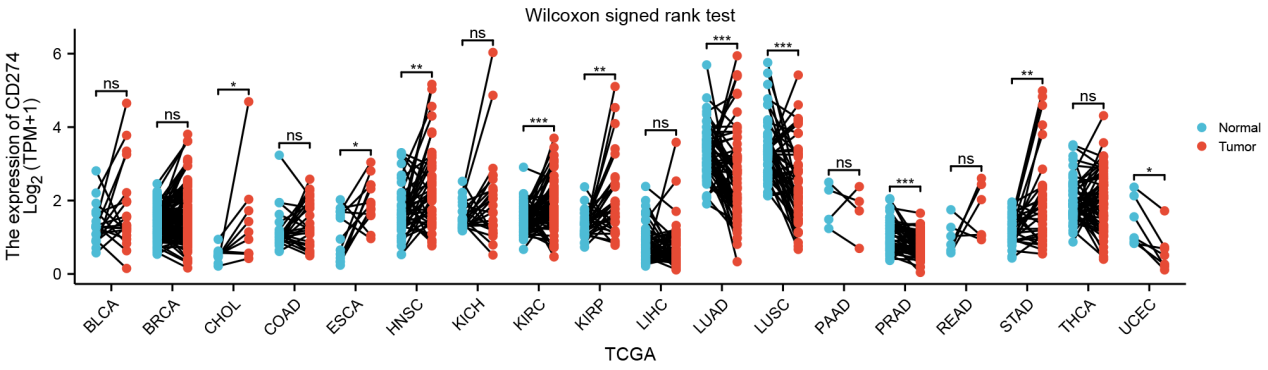
**

Supplementary Fig. S1 T test of *CD274* differential expression between tumor tissues and normal tissues in LUSC, HNSC, UCEC, PRAD, BLCA, CHOL, BRCA, ESCA, KICH, KIRP, LUAD, STAD, PRAD, READ, THCA, LIHC, COAD, and KIRC. * *P* < 0.05; ** *P* < 0.01; *** *P* < 0.001.


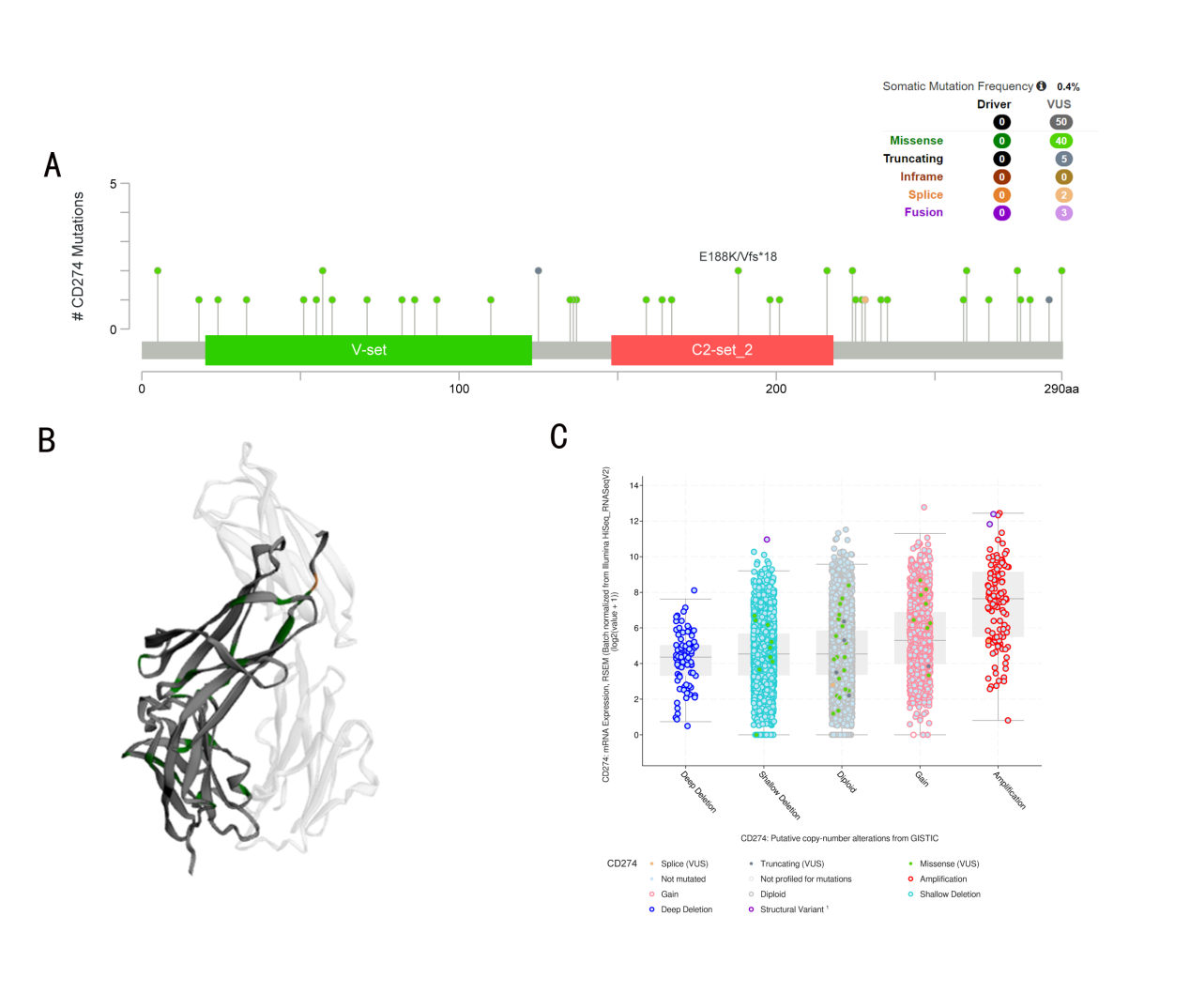


Supplementary Fig. S2 Mutation of *CD274.* The site, type, and number of mutations in the *CD274* gene (A)*.* The E188K/Vfs*18 mutation site in the three-dimensional protein structure of *CD274* (B). *CD274*’s alteration types in pan-cancer (C).


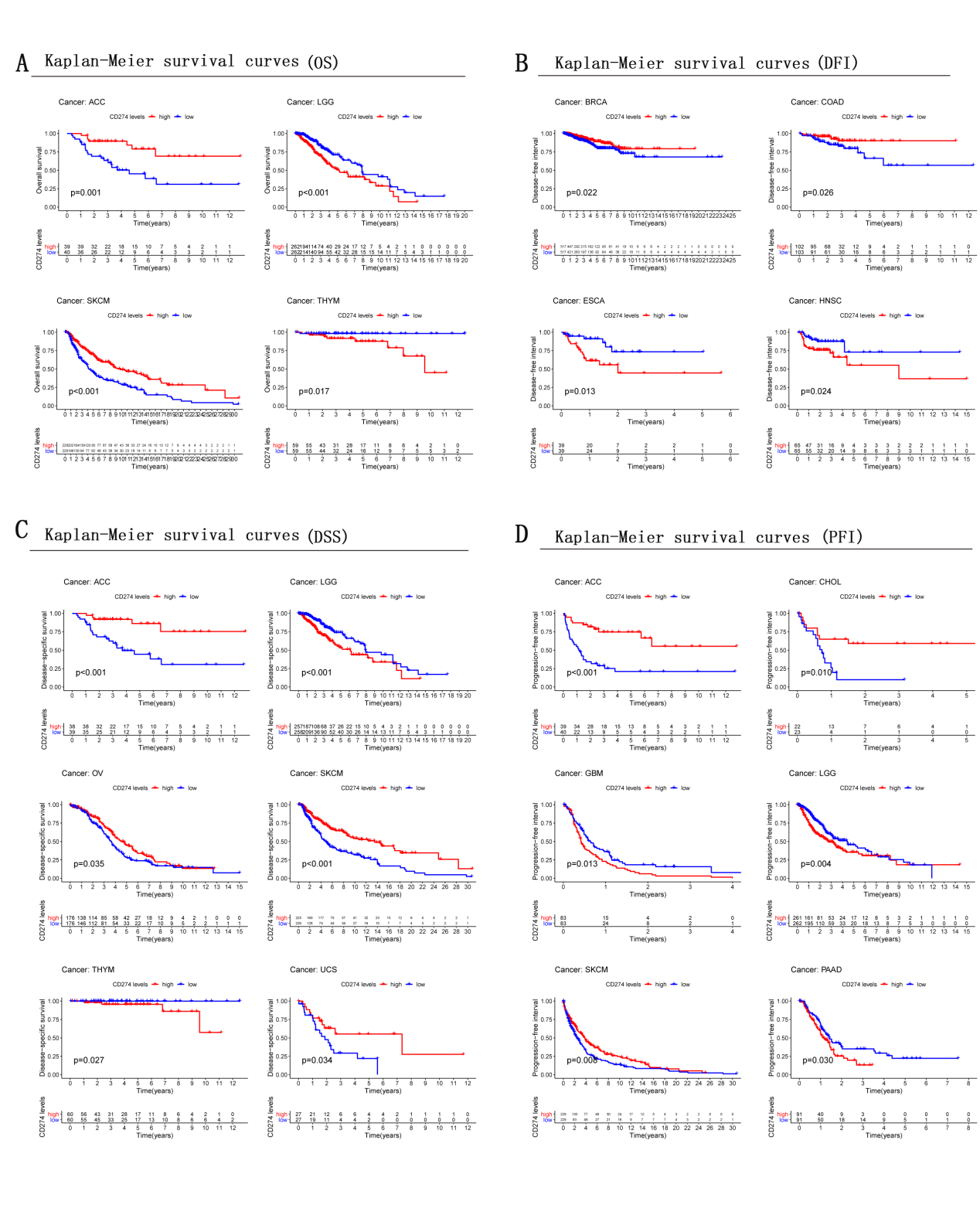


Supplementary Fig. S3 Correlation between *CD274* expression level and patients’ survival level. K-M survival curves for ACC, LGG, SKCM, and THYM’s OS (A), BRCA, COAD, ESCA, and HNSC’s DSS (B), ACC, LGG, OV, SKCM, THYM, and UCS’s DFI (C), and ACC, CHOL, GBM, LGG, SKCM, and PAAD’s PFI (D).


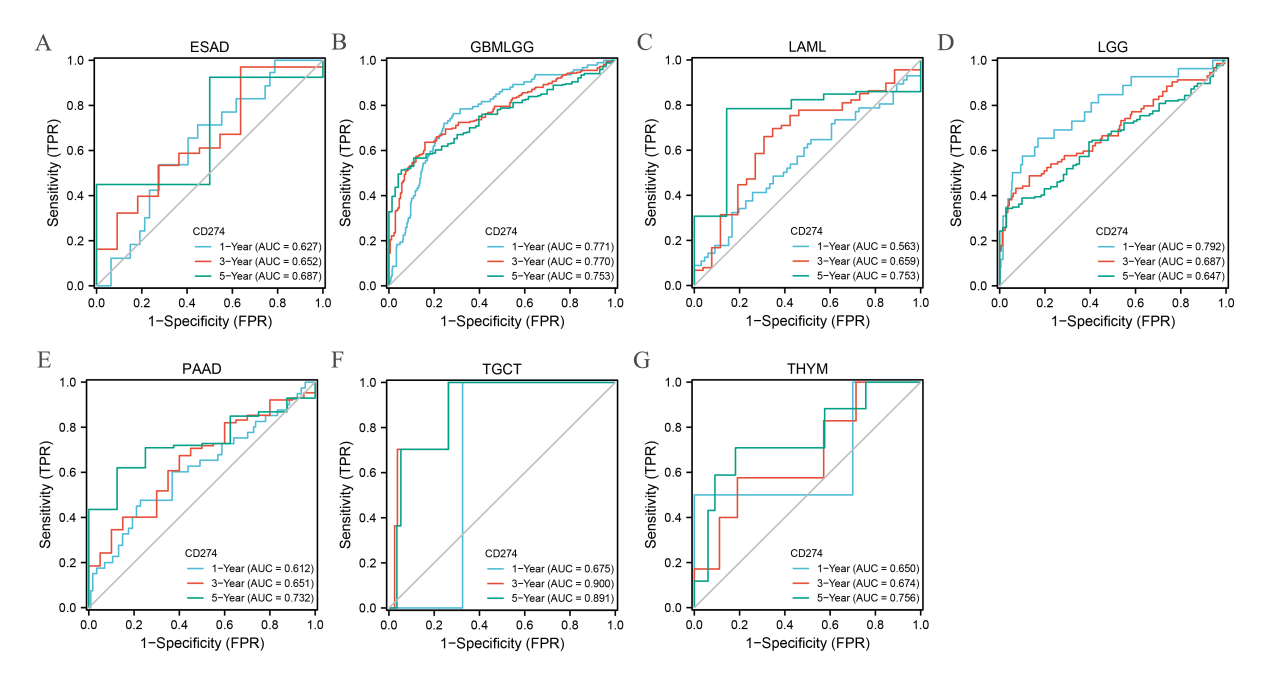


Supplementary Fig. S4 ROC curves (1-Year, 3-Year, and 5-Year) of ESAD, GBMLGG, LAML, LGG, PAAD, TGCT, and THYM in accordance with *CD274*-derived risk score.


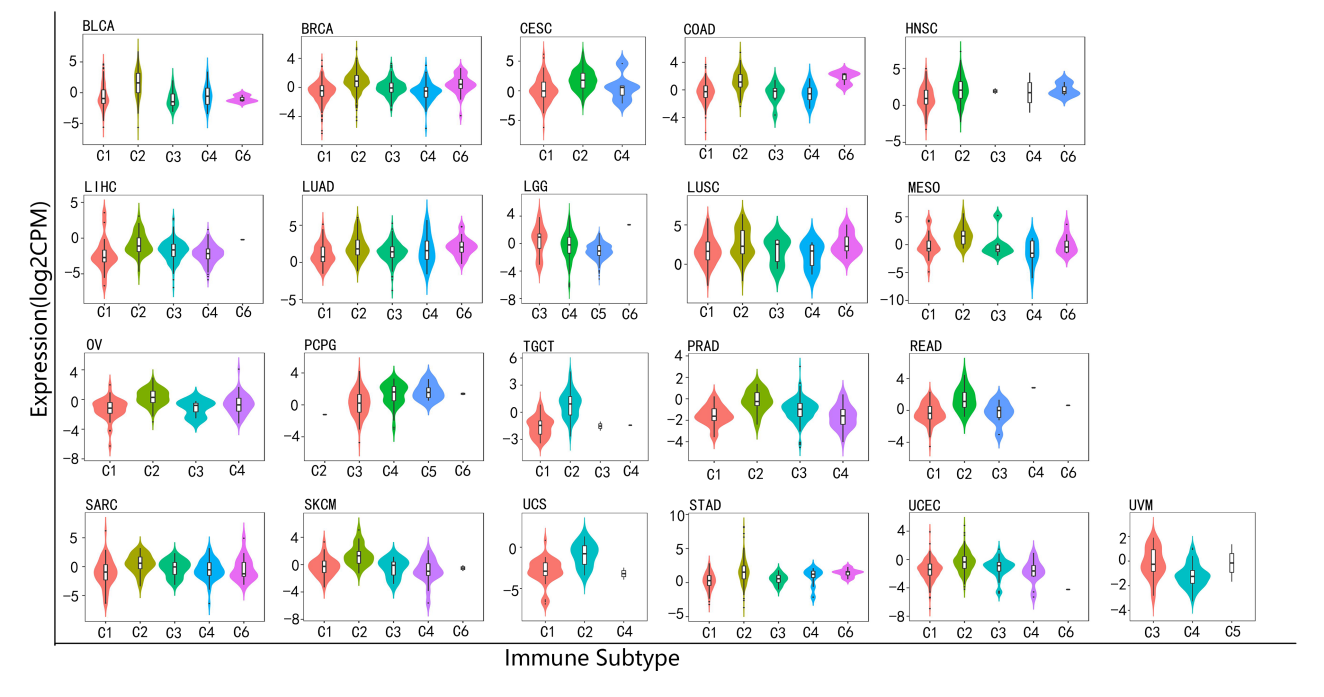


Supplementary Fig. S5 *CD274* expression in immune subtypes in LUAD, BLCA, CECS, BRCA, COAD, HNSC, LIHC, LUSC, MESO, PCPG, PRAD, OV, READ, SARC, SKCM, STAD, LGG, TGCT, UCS, UCEC, and UVM based on TISDB.


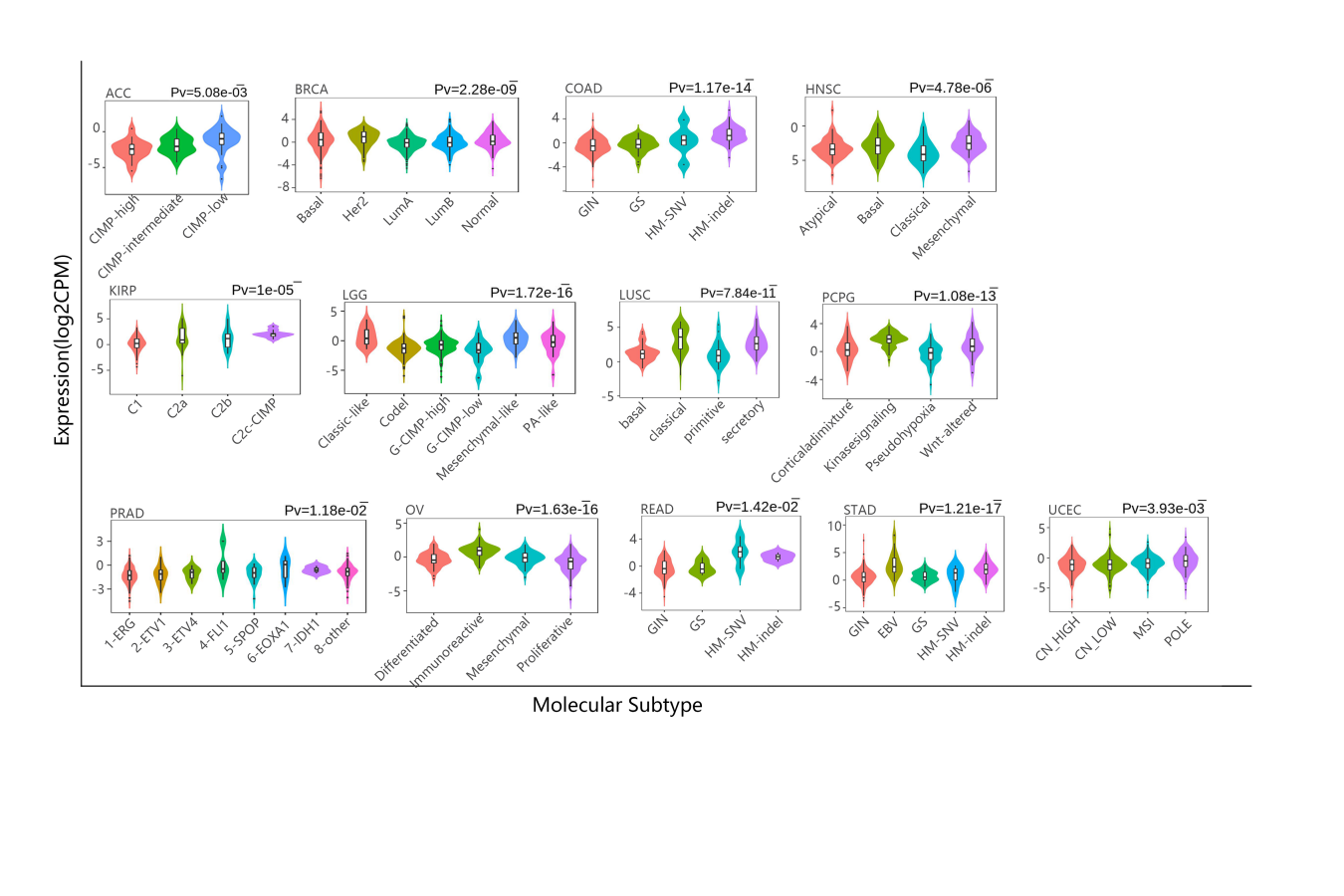


Supplementary Fig. S6 *CD274* expression in a variety of molecular subtypes of OV, HNSC, ACC, COAD, BRCA, KIRP, LUSC, PCPG, PRAD, READ, STAD LGG, and UCEC by TISDB analysis.


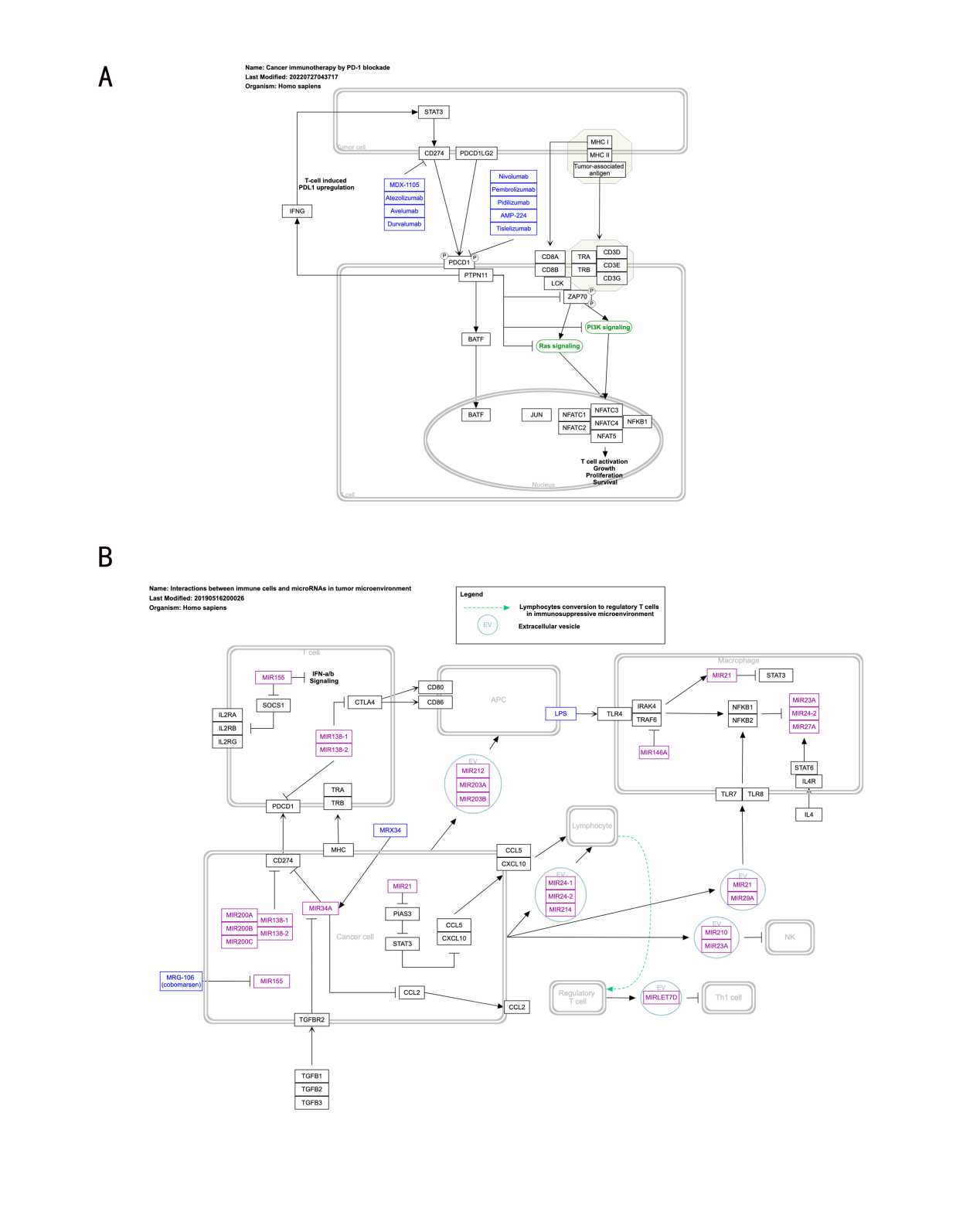


Supplementary Fig. S7 Significant biological pathways related to *CD274*. Cancer immunotherapy by *CD274* blockade (A). Interaction of immune cells and microRNAs in the tumor microenvironment (B).
